# Supplementary material for: Phylogenetic and population genetic analyses of Thrips tabaci Lindeman (Thysanoptera: Thripidae) on Allium host in India
Source: PeerJ. 2024 Jul 12;12:e17679. doi: 10.7717/peerj.17679 (PMC11249009; doi:10.7717/peerj.17679)
Supplement: Supplemental Information 1 [file peerj-12-17679-s001.docx]

**Supplementary Files**

**Figure 1: Detailed representation of SNP distribution in the mtCOI gene sequences of different thrips samples**


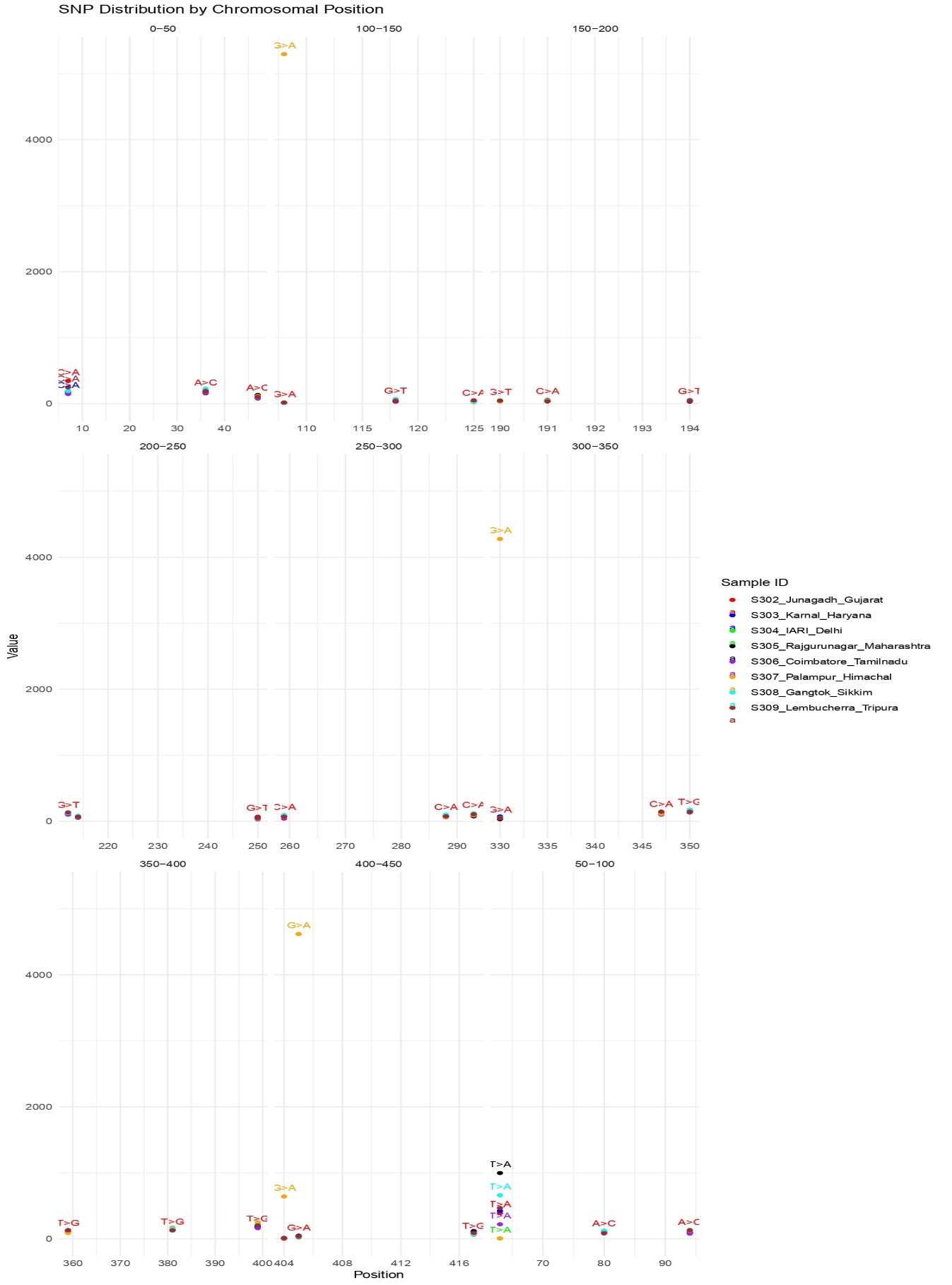


**Supplementary tables**

**Table 1: Pairwise Nm values between sub-populations of Group A**

|  | South | East | West | Northeast | Central |
| --- | --- | --- | --- | --- | --- |
| North | 4.04 | -84 | 3.24 | -84 | 4.04 |
| South |  | -12 | 120 | -8 | 5.71 |
| East |  |  | 3.24 | 12 | 3.53 |
| West |  |  |  | 12 | 12 |
| Northeast |  |  |  |  | 1 |

**Table 2: Pairwise Nm values between sub-populations of Group B**

|  | South | East | West |
| --- | --- | --- | --- |
| North | 8.56 | 2.59 | 0.82 |
| South |  | 4.54 | 1.91 |
| East |  |  | 3.36 |

**Table 3: Pairwise Fst values between sub-populations of Group A**

|  | South | East | North | Northeast | Central |
| --- | --- | --- | --- | --- | --- |
| West | 0.14815 | 0 | 0.21982 | -0.00152 | 0 |
| South |  | 0.03774 | 0.21868 | -0.00168 | 0.22222 |
| East |  |  | 0.20427 | -0.0106 | 0 |
| North |  |  |  | -0.35084 | 0.22151 |
| Northeast |  |  |  |  | 0 |

**Table 4: Pairwise Fst values between sub-populations of Group A**

|  | South | East | West |
| --- | --- | --- | --- |
| North | -0.01817 | 0.19318 | 0.66698 |
| South |  | 0.00172 | 0.52185 |
| East |  |  | 0.67167 |
